# Network analysis of perception-action coupling in infants

**DOI:** 10.3389/fnhum.2014.00209

**Published:** 2014-04-08

**Authors:** Naama Rotem-Kohavi, Courtney G. E. Hilderman, Aiping Liu, Nadia Makan, Jane Z. Wang, Naznin Virji-Babul

**Affiliations:** ^1^Neuroscience Program, University of British ColumbiaVancouver, BC, Canada; ^2^Department of Physical Therapy, University of British ColumbiaVancouver, BC, Canada; ^3^Electrical and Computer Engineering, University of British ColumbiaVancouver, BC, Canada; ^4^Department of Developmental Neurosciences and Child Health, Child and Family Research InstituteVancouver, BC, Canada

**Keywords:** infant, functional connectivity, action perception, EEG, graph theory, motor experience, mirror neuron system

## Abstract

The functional networks that support action observation are of great interest in understanding the development of social cognition and motor learning. How infants learn to represent and understand the world around them remains one of the most intriguing questions in developmental cognitive neuroscience. Recently, mathematical measures derived from graph theory have been used to study connectivity networks in the developing brain. Thus far, this type of analysis in infancy has only been applied to the resting state. In this study, we recorded electroencephalography (EEG) from infants (ages 4–11 months of age) and adults while they observed three types of actions: (a) reaching for an object; (b) walking; and (c) object motion. Graph theory based analysis was applied to these data to evaluate changes in brain networks. Global metrics that provide measures of the structural properties of the network (characteristic path, density, global efficiency, and modularity) were calculated for each group and for each condition. We found statistically significant differences in measures for the observation of walking condition only. Specifically, in comparison to adults, infants showed increased density and global efficiency in combination with decreased modularity during observation of an action that is not within their motor repertoire (i.e., independent walking), suggesting a less structured organization. There were no group differences in global metric measures for observation of object motion or for observation of actions that are within the repertoire of infants (i.e., reaching). These preliminary results suggest that infants and adults may share a basic functional network for action observation that is sculpted by experience. Motor experience may lead to a shift towards a more efficient functional network.

Long before an infant can reach for his favorite snack, he observes his mother reaching for a glass of water. How does he perceive this action? Will his perception change as he gains experience in reaching and grasping his own desired objects? Although the infant has not yet experienced the motion of grasping, observing his mother’s actions may be priming his neural pathways towards performing the same action. A growing body of research suggests a strong link between perception and action, and has revealed a network of brain regions that are activated during both the observation and execution of movement (Rizzolatti and Luppino, 2001). The neurological network supporting this activation is thought to be mediated by the mirror neuron system, which was originally described in monkeys (Rizzolatti and Luppino, 2001). This system includes neurons in the ventral premotor cortex, inferior parietal lobe and the superior temporal sulcus (Rizzolatti and Craighero, 2004).

The mirror neuron system is thought to play a fundamental role in both action observation and imitation (Rizzolatti and Craighero, 2004). Observed actions are processed as both visual events that can be perceptually described and recognized, as well as motor events that are represented as a sequence of motor commands, and that can be learned and replicated (Grossmann et al., 2007). These two processes can be engaged simultaneously and information may be exchanged between them during action recognition, prediction, and perception (Keysers and Gazolla, 2007; Kilner et al., 2007). A number of studies have found indirect evidence for mirror neuron activity early in infancy, during action observation tasks using mu (8–13 Hz) suppression as an index of mirror neuron activity (Southgate et al., 2010; Virji-Babul et al., 2012; Grossmann et al., 2013; Warreyn et al., 2013). At rest, neurons in the sensorimotor area fire synchronously resulting in large amplitude electroencephalography (EEG) oscillations in the mu frequency band. When subjects execute, imagine, or observe movements, these neurons fire asynchronously decreasing the power of the mu band (Pfurtscheller and Neuper, 1997; Muthukumaraswamy et al., 2004). It has been hypothesized that the mu rhythm reflects downstream modulation of primary sensorimotor areas by mirror neuron activity, representing a critical information processing function: translating perception into action (Pineda, 2005). Infancy provides a window to investigate the role of action observation in action production, since many new motor skills are acquired during this period. Marshall et al. (2011) studied EEG desynchronization of the mu rhythm in 14-month-old infants during action observation and during action performance. They reported that desynchronization during action performance was restricted to central electrode sites, whereas action observation was associated with a broader desynchronization across frontal, central and parietal sites. This pattern of activation suggests that the infant central rhythm shares functional properties with the adult mu rhythm. However, they reported that the infant responses were characterized by a smaller magnitude of EEG desynchronization in comparison to the adults. Two recent studies using electromyography (EMG) recordings during an action observation paradigm suggest that perception-action coupling occurs gradually during the first year of life. While 6 month old infants show evidence of motor resonance only when an action goal is achieved, 9 month old infants anticipate the action goal and demonstrate early EMG activity (Turati et al., 2013; Natale et al., 2014). These results suggest that the first year of life is characterized by critical development changes in perception-action coupling. However, our understanding of how motor experience influences this coupling is still limited. We recently examined the EEG response of young infants while observing three types of actions: (a) reaching for an object; (b) independent walking; and (c) object motion. We showed that young infants had significant motor resonance to all types of actions in the sensorimotor regions. Only observation of human goal-directed actions led to significant responses in the parietal regions. Importantly, there was no significant mu desychronization observed in the temporal regions under any observation condition. In addition, the onset of mu desynchronization occurred earliest in response to object motion, followed by reaching, and finally walking. Our results suggest that the infants may have a basic, experience-independent sensorimotor mechanism optimized to detect all coherent motion that is modulated by experience (Virji-Babul et al., 2012). We now extend this work to specifically examine the functional brain networks underlying action observation using graph theory based analysis.

Graph theory allows the quantitative analysis of network organization, characterizing the brain as sets of networks. Each network is comprised of nodes that represent distinct brain regions, and edges that delineate pathways connecting these regions. The relationship between nodes and edges provides information about the organization and efficiency of the network. Networks with an ordered structure have a high clustering coefficient (a measure that depicts the connectedness of immediate neighbors around individual vertices), long characteristic path length (an index reflecting the overall integration of the network), low global efficiency (defined as the average inverse shortest path) and low density or cost (the fraction of present connections to possible connections) (Wu et al., 2012a). In contrast, randomly organized networks are characterized by a low clustering coefficient, short characteristic path length, and a high global efficiency and density. Combining ordered networks with a certain fraction of randomly rewired links will yield “small-world” networks, with cohesive neighborhoods, short characteristic path length, and a high global efficiency (Wu et al., 2012a). Watts and Strogatz (1998) suggest that small-world networks, which balance between local specialization and global integration, are optimal for information processing. They further showed that several real-life networks possess small-world features. Recent studies have investigated the topological organization and structure of functional and dysfunctional adult brain networks by means of graph theory (Achard and Bullmore, 2007; Wu et al., 2012b, 2013); however, relatively few studies have investigated the functional connectivity of the infant brain. Fransson et al. (2010) were the first to investigate the functional architecture of the infant brain using resting state (rs)-fMRI. They reported that functional networks observed during sleep have small-world topology and that the cortical hubs in the newborn brain are predominantly confined to the primary sensory and motor regions of the brain. The authors hypothesize that the functional networks are already present, even if in rudimentary form, at birth and are organized primarily to support sensorimotor development. Given that sensorimotor networks are already present early in infancy, the question of how motor experience modulates properties of these networks is of great interest.

In the present study, we re-analyzed the EEG data from our previous study where infants observed three types of actions: actions that are developmentally within the motor repertoire of infants (i.e., reaching), actions that are developmentally not within the motor repertoire of infants (i.e., independent walking), and moving inanimate objects (e.g., a rolling ball). We compared responses between infants and adults and ask: (a) what are the characteristics of functional networks underlying action observation in infants and adults and (b) does motor experience modulate the characteristics of these networks during development? By using graph theoretical analysis, we characterized the topological differences between the emerging (infant) and mature (adult) action observation systems.

## Methods

### Participants

Initially, 14 infants were recruited for this study; however four infants were excluded from analysis due to movement or insufficient artifact-free trials per condition during data collection. Therefore, a total of 10 infants between the ages of 4 and 11 months (mean age: 6.94 months, *SD* = 2.35, 6 males, 4 females) were included in our analysis. Parents provided information about the reaching and ambulatory experience of their infant. All infants were able to perform a reaching motion but none had started to walk independently at the time of the experiment according to parent reports. Parents provided written consent according to the guidelines specified by the Human Ethics Review Board at the University of British Columbia.

Initially, 15 adults were recruited for this study, however four adults were excluded from analysis due to lack of sustained attention to stimuli during data collection. Therefore, a total of 11 adults between the ages of 20 and 50 years (mean age: 31 years, *SD* = 11.72, 8 females, 3 males) were included in our analysis. All adults provided written consent according to the guidelines specified by the Human Ethics Review Board at the University of British Columbia. All subjects reported normal or corrected-to-normal vision.

### Stimuli

**Figure 1:**
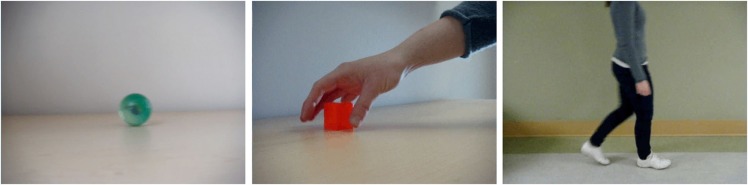
**Screenshot examples for the three different conditions (from left to right object movement, reaching for an object, walking)**.

Videos of 1.5-s duration depicting three different actions: human walking, hand reaching for objects, and object motion were prepared. The object motion videos displayed five different toys, and included either a ball (3 videos) or a toy car (2 videos) rolling across a surface. The videos were randomly selected for presentation by the computer program “Presentation”. Adult actors were used for the reaching and walking videos. Videos were recorded against a neutral background. Unlike previous studies, we did not show the face of the actors in any of the displays. A total of 60 videos (20 walking, 20 reaching, and 20 object motions) were included, and were presented in a random order (Screenshot examples are presented in Figure 1). Each video was followed by a 500 ms of blank screen and the total viewing time was 120-s. All adults and infants were shown the same number of videos (total of 60).

### Experimental setup and procedure

All adult participants were seated on a chair in front of a 112 cm (diagonal length) projector screen at a viewing distance of approximately 190 cm. Infants were seated on their parent’s lap who were sitting on a chair at a distance of approximately 190 cm. The figures used for the adults and the infants were the same. For walking motion, the figure from the feet to the trunk measured on the screen 42 cm in height and 14 cm in width. For reaching motion, the visible part of the arm, starting at the tip of the finger, measured on the screen 84 cm in height and 19 cm in width. The cube that the arm reaches for measured 12 cm in height and 12 cm in width. The diameter of the ball used in the object motion measured 13 cm on the screen. A camera was placed below the projection screen to monitor the participants’ eye and limb movements. Only trials with no limb movement and during which the participant observed the video displayed were included in the analysis.

### EEG recording and analysis

EEG was recorded using a 64-channel Hydrogel Geodesic Sensor-Net (EGI, Eugene, OR). EEG was recorded with a Net Amps 300 amplifier at a sampling rate of 250 Hz. Scalp electrode impedances were usually less than 50 kΩ. The signal was collected referenced to the vertex (Cz). The signal was then filtered from 4 to 40 Hz, and a notch filter of 60 Hz was included. The EEG signals were interpolated at 27 locations on the scalp using BESA’s Virtual Standard 10-10 Average montage.

### Brain connectivity network modeling

In this paper, the false discovery rate controlled PC (PCFDR) algorithm (Li and Wang, 2009) was used to compute brain connectivity networks. PCFDR is a computational method that is based on the error rate criterion of the discovered network. It estimates the ratio of the falsely detected connections to all those detected. This method is capable of asymptotically controlling the false discovery rate (FDR) under the predefined levels. In comparison with the traditional Type-1 and Type-2 error rates, FDR has more reasonable error rate criteria in brain connectivity modeling since it is directly related to the uncertainty of the networks that are studied. The details and pseudo-code of the PCFDR algorithm are described in Li and Wang (2009). The FDR threshold was set at the 5% level. The binary undirected connectivity networks were computed for each individual subject and each condition independently.

### Graph Theoretical Analysis

We used graph theoretical based analysis to extract the structural features from the learned networks (Bullmore and Sporns, 2009). Here we utilized traditional graph theoretical measures to characterize the network features in terms of density, global efficiency and modularity. Density is defined as the fraction of present connections to all possible connections. Global efficiency describes the communication ability of the entire graph (Latora and Marchioro, 2001) and is defined as the average of the inverse shortest path. Modularity of the network is used to measure how well the network can be divided into sub-modules (Newman, 2004). A higher value of modularity demonstrates that the graph is more clustered with tighter connections within modules. We used the Brain Connectivity Toolbox (Rubinov and Sporns, 2010) running Matlab (Natick, MA) to perform the graph theoretical analysis.

## Results

**Figure 2:**
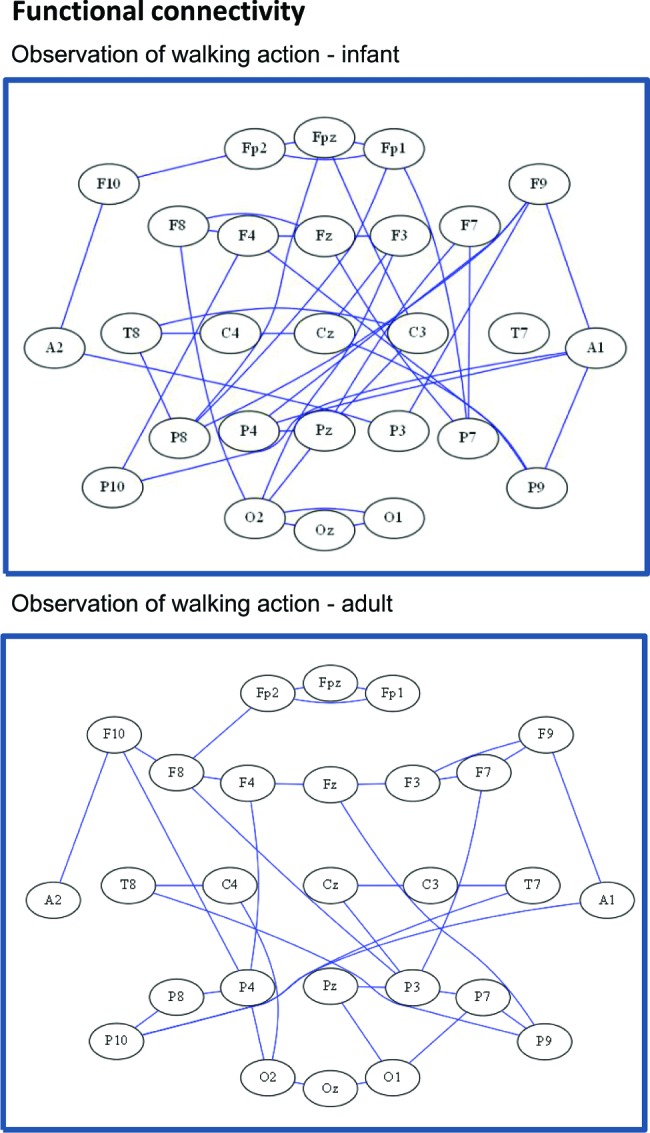
**Representative functional connectivity headplot graphs from both an infant subject (top), and an adult subject (bottom), for the walking condition demonstrating differences in the topological organization of the network**.

Qualitatively, the overall network functional connectivity for the infants and the adults were similar for the observation of object and reaching conditions. In contrast, there were subtle differences visible between the infant and adult graphs in the observation of walking condition. Figure 2 shows the functional connectivity graphs from one infant (top panel) and one adult subject (bottom panel) for the walking condition. Note that the infant’s graph appears to have denser connections and is less clustered than the adult’s graph.

**Figure 3:**
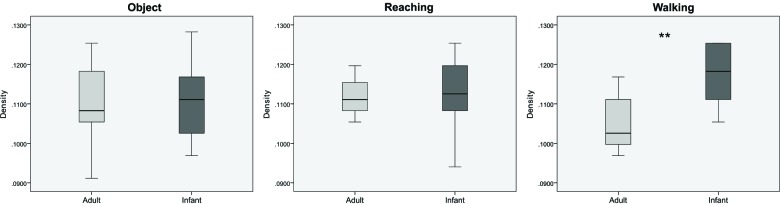
**Boxplots showing differences between infants and adults in mean values of density for the three conditions (from left to right: object, reaching, walking)**. ** *p* < 0.01. Whiskers are representing minimum and maximum points of the data.

We then computed the overall measures of density, global efficiency, and modularity in each group. Figure 3 shows the differences in density between the groups, in each condition. A mixed ANOVA showed a statistically significant interaction between age and condition for density (*F*_(2, 38)_ = 3.664, *p* = 0.035, *η*^2^ = 0.162). Follow up testing showed statistically significant higher density values for the walking condition for the infants in comparison with adults (*F*_(1, 19)_ = 12.249, *p* = 0.002, *η*^2^ = 0.392). There were no significant differences for the object and reaching conditions (*F*_(1,19)_ = 0.03, *p* = 0.863, *η*^2^ = 0.002, and *F*_(1,19)_ = 0.213, *p* = 0.650, *η*^2^ = 0.011 respectively).

**Figure 4:**
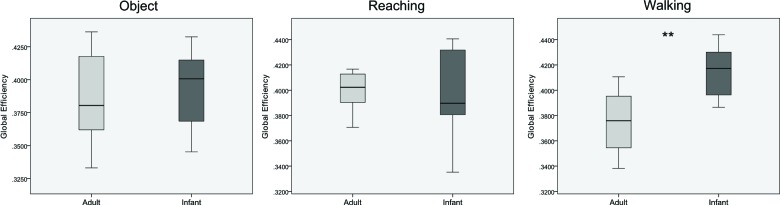
**Boxplots showing differences between infants and adults in mean values of global efficiency for the three conditions (from left to right: object, reaching, walking)**. ** *p* < 0.01. Whiskers are representing minimum and maximum points of the data.

Figure 4 shows the differences in global efficiency between the groups across the three conditions. A mixed ANOVA showed a statistically significant interaction between age and condition (*F*_(2, 38)_ = 4.857, *p* = 0.013, *η*^2^ = 0.204). Follow up testing revealed statistically significant higher global efficiency values for infants in comparison with adults for the walking condition (*F*_(1, 19)_ = 14.719, *p* = 0.001, *η*^2^ = 0.437), with no significant differences for the object and reaching conditions (*F*_(1,19)_ = 0.131, *p* = 0.721, *η*^2^ = 0.007, and *F*_(1, 19)_ = 0.013, *p* = 0.910, *η*^2^ = 0.001 respectively).

**Figure 5:**
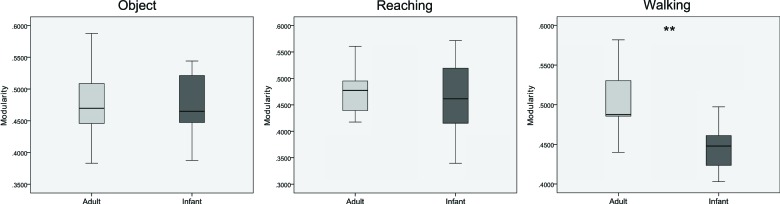
**Boxplots showing differences between infants and adults in mean values of modularity for the three conditions (from left to right: object, reaching, walking)**. ** *p* < 0.01. Whiskers are representing minimum and maximum points of the data.

Differences in modularity between the groups across the three conditions are shown in Figure 5. A one-way ANOVA for each condition revealed statistically significant lower modularity values for the infant group in comparison with the adult group for the walking condition (*F*_(1, 19)_ = 11.027, *p* = 0.004, *η*^2^ = 0.367). There were no significant differences for the object and reaching conditions (*F*_(1,19)_ = 0.016, *p* = 0.900, *η*^2^ = 0.001, and *F*_(1,19)_ = 0.098, *p* = 0.758, *η*^2^ = 0.005 respectively). There were no main effects nor interactions between age and condition for modularity when tested with mixed ANOVA (*F*_(2, 38)_ = 2.777, *p* = 0.075, *η*^2^ = 0.128).

## Discussion

In the current paper we used graph theoretical analysis to investigate EEG-based functional brain networks in relation to action observation in both infants and adults. Previous studies have studied brain organization in infants who are sleeping. These foundational studies have been critical in establishing and modeling complex brain networks, demonstrating that a basic functional network with small world features is present at birth (Fransson et al., 2010; Supekar et al., 2010; Vogel et al., 2010). However, to our knowledge the functional connectivity of the infant network in conditions other than sleep—i.e., under a task condition, has not been studied. Our study provides preliminary data characterizing the topology of infant networks during an active, visual-perceptual task and offers new insights into the potential role of motor experience in shaping the networks to a mature form.

In our study we found that the adult and infant networks were comparable in terms of organization and global network efficiency in response to observing experienced motion (i.e., reaching) and object motion. Interestingly, however, the infant networks in response to observing non-experienced motion (i.e., independent walking) were much less structured compared to that of the adults. These preliminary results suggest that motor experience may in fact, play a role in the development of the action-perception network, triggering the network to evolve towards a more efficient configuration. This evolution may be reflective of changes occurring at a structural neural level during which inefficient or excess connections are pruned and sculpted by experience to result in a more efficient organization (Boersma et al., 2011).

Changes in the structure of brain networks during development have been reported recently. For example, Fan et al. (2011) recently reported changes in brain networks in relation to efficiency and modular organization from birth to 2 years of age. They showed that from the age of 1 month to 2 years, as the brain develops, modularity increases. Moreover, the modular assignment for brain regions changes dramatically, reflecting dynamic anatomical segregation and integration of brain regions. Similarly, Khundrakpam et al. (2012) examined the developmental changes in the organization of structural brain networks from early childhood to late adolescence in the Pediatric MRI Data Repository. They found that global efficiency increased from early to late childhood and decreased from late childhood to late adolescence. Modularity was reduced for the late childhood group in comparison to the other groups. This indicates that structural brain networks may be less organized during early development and progress to a more optimal configuration during adolescence. Our results provide further support to these findings, demonstrating a possible link between motor experience and changes in network efficiency.

There are of course, differences in the types of actions that the infants observed that could have led to subtle changes in network organization. For example, the reaching action shows a goal-directed, transitive movement and displays only one body part, whereas the walking motion demonstrates an intra-transitive movement and displays an adult actor’s lower limbs and trunk. These differences may influence the mapping of actions to an individual’s body schema however we would expect the effects to reflect a systematic shift across both groups. Therefore, we propose that the significant differences between groups in our study are likely due to the presence or absence of motor experience with the walking motion. Several studies have investigated the interaction of previous motor and visual experience in modulating the action observation network. For example, Calvo-Merino et al. (2005) investigated the neural responses of experts in classical ballet and capoeira compared to non-expert control subjects, and found increased activity in the action observation network when subjects observed movements in the style of which they were trained. Jola et al. (2012) compared the corticospinal excitability of subjects who frequently watched ballet or Indian dance with subjects who had no experience in watching dance. They reported that corticospinal excitability was highly correlated with previous experience of watching specific dance forms. In order to determine the relative contribution of motor and visual experience Calvo-Merino et al. (2006) found, that the action observation network relies more on the motoric experience of dance experts rather than visual experience. Together these studies highlight that individual motor experience, may be correlated with the magnitude of response in the action observation network.

Our understanding of how the networks underlying action perception are related to the networks underlying action execution during infancy remains elusive. Shimada and Hiraki (2006) proposed that early in life, infants may display a broadband response to human motion and all coherent motion. This predisposition to understanding object and human motion may be refined with experience through a process of Hebbian learning (Del Giudice et al., 2009), providing a mechanism for the integration of perceptual-motor learning. Nagai et al. (2011) further proposed a computational model of the development of the action perception system in which they suggest that there may be a correlation between the development of visual perception and sensorimotor development. In their model, they show that in the early stages of development, all motion is perceived and processed at a very basic level; as the spatiotemporal resolution of vision develops, their proposed model begins to discriminate between motions of the self and the motions of others. Through feedback and sensorimotor learning, an association is created between the motor commands of the self and the motions of others. These modeling results provide a theoretical basis of how perceptual-motor coupling may develop in infants and offers directions for future research.

### Limitations

Our study was limited by the difficulty in recruiting infants that were able to attend to the stimuli during EEG data collection without moving. Recruitment challenges required us to collect data on a wider age range of infants than would be ideal. This limitation may have reduced the power of statistical significance in functional connectivity differences due to the variability in motor experiences within the infant group (i.e., the older infants likely have more motor experience with reaching than the younger infants). In addition, although none of the infants had experience in walking independently, older infants may have started cruising or taking a few independent steps. We did not collect detailed information on whether the infants had begun to take a few independent steps and it is possible that this variability may have also led to some minor differences in functional connectivity. However, this is unlikely as our previously published data (Virji-Babul et al., 2012) clearly showed differences in the magnitude of mu desynchronization as well as the onset of this activity in relation to the three conditions. The onset of mu activity was slowest for the walking condition and in addition there were significant differences in the time frequency distributions for the walking condition. Based on these previously published data, we feel that the current analysis provides further evidence that observation of walking is in fact, processed differently from both object and reaching motions. A larger sample would allow comparisons between groups with similar motor experiences and provide more insights into the effect of experience on motor perception. Another limitation of our study is the lack of an action execution condition. A direct comparison of action execution with action observation using a graph theory approach would help differentiate the perception-action coupling networks with the action execution networks and would significantly advance our understanding of the fundamental mechanisms underlying the development of the perceptual motor system.

### Summary and future directions

Using high-density EEG measurements and utilizing graph theory based analysis techniques, we provide preliminary results that suggest motor experience may play a role in the structural organization of brain networks. Early in infancy, the basic brain networks for perception-action coupling appear to have been established. The differences in the organization of these basic networks seem to be associated with previous motor experience: actions related to object motion and goal directed upper limb motions that the infants could already perform showed a more adult-like organization; in contrast, actions in which the infants had little or no previous motor experience showed a more random organization, suggesting that motor experience may play a key role in developing more cost-effective networks. These preliminary results warrant further exploration with more subjects to examine the relationship of motor experience and functional network development in infants. For example, comparing the networks of young infants who are not yet walking independently to older infants who have recently begun to walk independently could provide additional insights into the development of action observation network connectivity. In addition, comparing the network connectivity of a range of developmental motor behaviors both within the infants’ repertoire (i.e., crawling) and novel actions that are not within the infants’ motor repertoire (i.e., jumping) will help elucidate the influence of motor experience. Further research should investigate the role of motor experience throughout infancy, childhood, and adolescence in shaping functional connectivity.

## Conflict of interest statement

The authors declare that the research was conducted in the absence of any commercial or financial relationships that could be construed as a potential conflict of interest.
